# Quantitative end-tidal CO_2_ can predict increase in heart rate during infant cardiopulmonary resuscitation

**DOI:** 10.1016/j.heliyon.2019.e01871

**Published:** 2019-06-12

**Authors:** Christina N. Stine, Josh Koch, L. Steven Brown, Lina Chalak, Vishal Kapadia, Myra H. Wyckoff

**Affiliations:** a Central Ohio Newborn Medicine, Columbus, OH, USA; b Department of Pediatrics, The University of Texas Southwestern Medical Center, Dallas, TX, USA; c Department of Health System Research, Parkland Memorial Hospital, Dallas, TX, USA; d Division of Cardiac Critical Care, Phoenix Children's Hospital, Phoenix, AZ, USA

**Keywords:** Emergency medicine, ETCO2, Neonatal resuscitation, Infant resuscitation

## Abstract

**Aim:**

To determine the end-tidal CO_2_ (ETCO_2_) value that predicts a HR > 60 beats per minute (bpm) with the best sensitivity and specificity during neonatal/infant cardiopulmonary resuscitation (CPR) defined as chest compressions ± epinephrine in neonates/infants admitted to a CVICU/PICU.

**Methods:**

This was a retrospective cohort study from 1/1/08 to 12/31/12 of all infants ≤6 month of age who received CPR and had ETCO_2_ documented during serial resuscitations in the pediatric (PICU) or pediatric cardiovascular intensive care units (CVICU) of Children's Medical Center of Dallas. A receiver operator characteristic (ROC) curve was generated to determine the ETCO_2_ cut-off with the best sensitivity and specificity for predicting HR > 60 bpm. Each ETCO_2_ value was correlated to the infant's HR at that specific time.

**Results:**

CPR was provided for 165 infants of which 49 infants had quantitative ETCO_2_ documented so only these infants were included. The majority were in the CVICU (81%) and intubated (84%). Mean gestational age was 36 ± 3 weeks and median age (interquartile range) at time of CPR was 30 (16–96) days. An ETCO_2_ between 17 and 18 mmHg correlated with the highest sensitivity and specificity for return of a HR > 60 bpm. Area under the curve for the ROC is 0.835.

**Conclusions:**

This study provides critical clinical information regarding correlation between ETCO_2_ values and an adequate rise in heart rate in neonates and young infants during CPR. Quantitative ETCO_2_ monitoring allows CPR to progress uninterrupted without need to pause to check heart rate every 60 seconds until the critical ETCO_2_ threshold is reached. Quantitative ETCO_2_ monitoring as an adjunct to cardiac monitoring during infant CPR might enhance perfusion and improve outcomes.

## 1 Introduction

Cardiovascular collapse during the neonatal period is most commonly due to asphyxia [1, 2]. Although cardiopulmonary resuscitation (CPR) in the delivery room and/or the neonatal intensive care unit (NICU) is rare, both have high rates of mortality and morbidity [3, 4, 5]. The high mortality and poor outcomes in survivors demonstrates the critical need to optimize neonatal/infant chest compression methods.

End-tidal carbon dioxide (ETCO_2_) monitoring is a non-invasive tool that predicts and demonstrates return of spontaneous circulation (ROSC) during experimental and human cardiac arrest [6, 7, 8, 9]. In adults, low ETCO_2_ levels indicate poor cardiac output, poor cardiac perfusion pressure and predict low rates of ROSC [7, 9, 10, 11, 12, 13, 14]. This appears to be true in adults regardless of the mode of cardiac arrest [15]. CO_2_ is produced by aerobic cellular metabolism and transported to the right heart, pumped into the lungs and released into the exhaled air where it is measured as ETCO_2_ [16]. CO_2_ production, alveolar ventilation, and pulmonary perfusion determine ETCO_2_. If two of the three are constant, changes in ETCO_2_ will reflect changes in the third. Thus, if ventilation is constant and CO_2_ production is assumed to be very low and constant, exhaled CO_2_ depends on pulmonary perfusion and therefore correlates with cardiac output [8, 17, 18]. In adults with loss of spontaneous circulation, there is a progressive decrease in ETCO_2_ with marked increases in ETCO_2_ to approximately 28 mmHg indicating ROSC during CPR [7, 10].

In neonatal CPR, compressions are continued until an audible heart rate >60 bpm is achieved. The ETCO_2_ value that correlates with HR > 60 bpm during CPR in infants is unknown. The objective of this study was to determine if there is a consistent ETCO_2_ threshold that correlates with return of an audible HR > 60 bpm following asystole or bradycardia during CPR in neonates/infants. If so, ETCO_2_ monitoring could limit unnecessary interruptions in compressions until the threshold is reached. Since infant CPR is such a rare event in the delivery room and NICU, we examined a population of infants <6 months that were in the Pediatric Intensive Care Unit (PICU) and Cardiovascular Intensive Care Unit (CVICU) to determine the ETCO_2_ value that indicates HR > 60 bpm during CPR.

## 2 Methods

This project was approved by the Institutional Review Board of the University of Texas Southwestern Medical Center. This retrospective cohort study was conducted at Children's Medical Center (CMC) of Dallas, TX, a regional pediatric tertiary care center. All patients who receive CPR at CMC are prospectively entered into the American Heart Association (AHA) Get with the Guidelines® Database. Study participants were infants less than 6 months of age who received cardiac compressions ± medications during their CMC hospitalization from 1-1–2008 to 12-31-2012 and had simultaneous ETCO_2_ and heart rate recorded by the medical team. ETCO_2_ was monitored using a Philips Microstream CO_2_ Extension (M3015A/M3015B, Philips Healthcare, The Netherlands). It is routine practice to monitor ETCO_2_ in all intubated infants in both of these units. Study subjects were initially identified through the Get with the Guidelines® database. Local data was extracted from this database and further chart review completed. The AHA Pediatric Advance Life Support (PALS) algorithm was used to resuscitate these infants. PALS recommends starting chest compressions for a heart rate or pulse less than 60 beats per minute (bpm). Other code medications such as epinephrine and atropine can be used depending on the reason for the code [19, 20]. ETCO_2_ data with correlated heart rate (usually taken off the monitor) was extracted from the code record. The presence or absence of a palpable pulse was not reliably recorded. Data recorded in addition to ETCO_2_ values were gestational age (GA) at birth, admission weight, weight at the time of CPR, as well as underlying medical diagnoses, ventilator support, medications, and vital signs just prior to and during CPR. The proposed mechanism for the cardiovascular collapse according to the primary medical team, and whether sustained ROSC ever achieved was also noted. ROSC was defined as return of spontaneous rhythm without need for extracorporeal membrane oxygenation.

### 2.1 Statistical analysis

Statistical analysis of the data was performed using SPSS (IBM SPSS, version 19). The median and mean were calculated for the patient groups for the individual data points. The sample size for this observational study was one of convenience and was 49, which represent all the infants who also had ETCO_2_ recorded during the resuscitation. If an infant had more than one CPR event, only the first event was included in the analysis. Infants that had congenital heart lesions and those with pulmonary hypertension were included. A receiver operator characteristic (ROC) curve was generated using ETCO_2_ values and heart rate values collected at the time of resuscitation for all infants. Each ETCO_2_ value and heart rate were used as a single data point. The data for the ROC curve did not use independent observations and individual bias may be present. A positive test was defined as an ETCO_2_ value associated with return of HR > 60 bpm.

### 2.2 Infants with decreased pulmonary blood flow

Infants with the potential for decreased pulmonary blood flow were included in the primary analysis but also were analyzed in a separate ROC curve. These infants were separated out based on the knowledge that ETCO_2_ can be affected by decreased pulmonary blood flow. The diagnoses included in this group were infants with pulmonary stenosis (n = 7), RV-PA conduit (n = 5), and BT shunt (n = 4). Infants with pulmonary hypertension (n = 5) were also included in this analysis since they also have the potential for decreased pulmonary blood flow. Inclusion in this group of infants was determined based on review of the cardiac lesions by Dr. J. Koch, pediatric cardiovascular intensivist.

## 3 Results

During the study period 349 infants received CPR in the PICU and CVICU. Of these infants, 165 were less than 6 months and 49 had at least one ETCO_2_ documented simultaneously with heart rate during resuscitation. Characteristics of the 49 infants are shown in Table 1.

**Table 1:** Infant characteristics.

Patient Characteristics	N = 49
OB EGA at birth (weeks)^a^	36 ± 3
Birth weight (kgs)^a^	2.7 ± 0.7
Current weight (kgs)^a^	3.7 ± 1.3
Age at time of CPR (days)^b^	30 (16–96)
Male (%)	28 (57%)
Potential for **↓** pulmonary blood flow	21 (43%)
Congenital Heart Disease (%)	40 (81%)
Potential for **↓** pulmonary blood flow due to cardiac anatomy (%)	20/40 (50%)
Intubated at time of CPR (%)	41 (84%)
Cause of cardiovascular collapse (%)	
Cardiac	27 (55%)
Respiratory	17 (35%)
Sepsis	3 (6%)
Non-accidental trauma	2 (4%)
ROSC achieved	37 (76%)
Number of codes while in hospital^b^	1 (1–2)

a mean ± standard deviation.

b median (interquartile range).

Twenty-one of these infants were classified with decreased pulmonary blood flow based on the patient's specific cardiac lesion or with the diagnosis of pulmonary hypertension. The highest cause of cardiovascular collapse in all infants was cardiac (55%) followed by respiratory (35%), sepsis (6%), and non-accidental trauma (4%). Sustained ROSC was achieved for 76% of the infants. For those infants with decreased pulmonary blood flow, the most common cause of cardiac collapse was respiratory (52%) and then cardiac (48%).

**Fig. 1:**
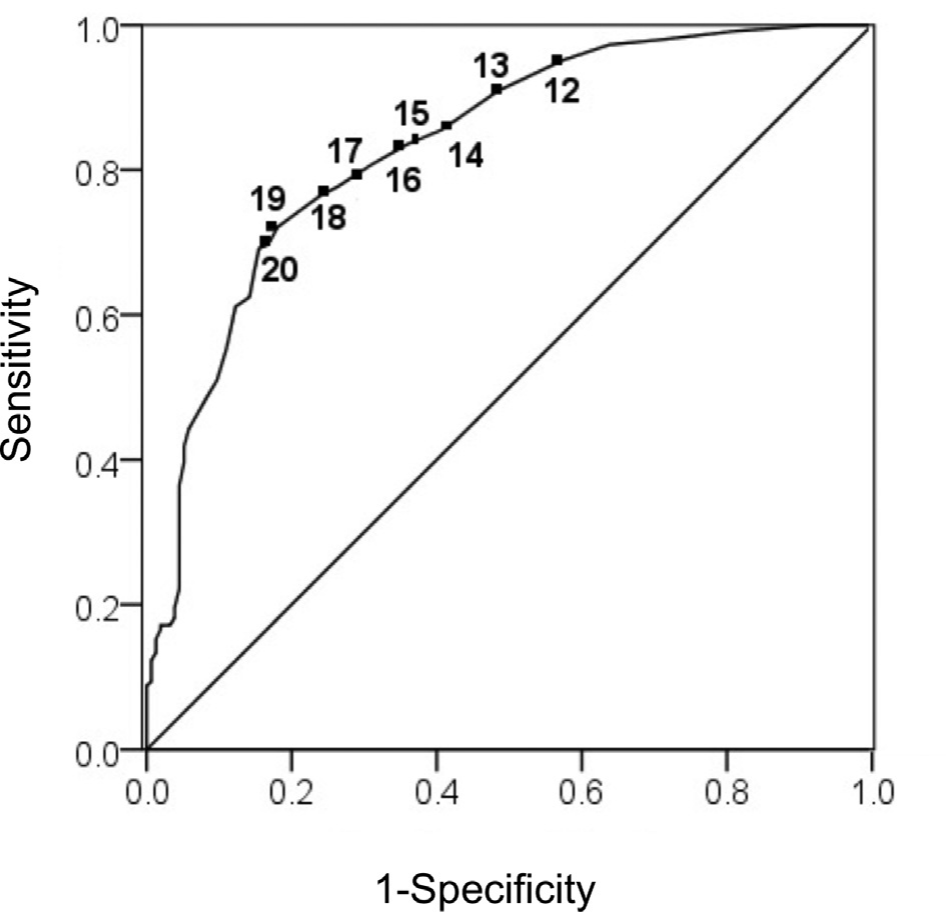
ROC curve for ETCO_2_ prediction of ROSC for all infants. The ETCO_2_ between 17 and 18 mmHg correlated with the highest sensitivity and specificity for return of a HR > 60 bpm. Area under the curve for the ROC is 0.835 with a p-value of less than 0.001. Positive likelihood ratio is 2.93 and 3.20 for ETCO_2_ of 17 mmHg and 18 mmHg respectively. PPV was 87.6% for ETCO_2_ of 17 mmHg and 88.5% for ETCO_2_ of 18 mmHg.

**Fig. 2:**
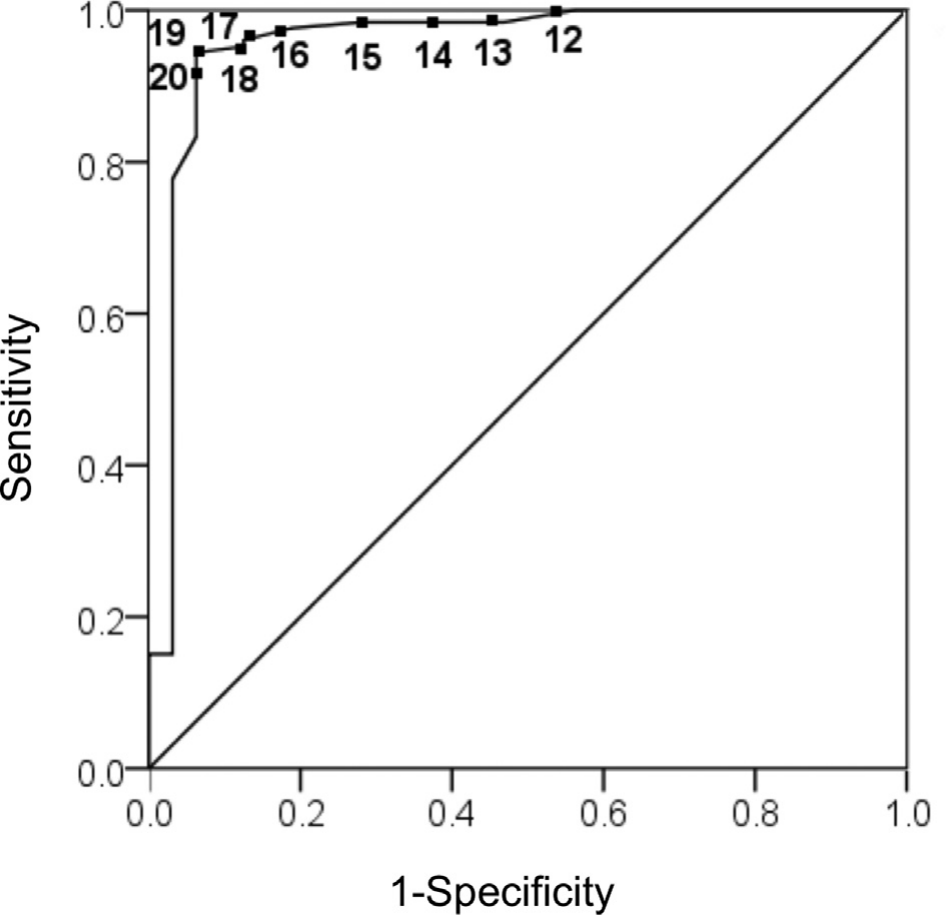
ROC curve for ETCO_2_ prediction of ROSC for infants with cardiac anatomy with potential for decreased pulmonary blood flow. ETCO_2_ of 17 was the predictor of HR > 60 with the highest sensitivity of 0.96 and specificity 0.88. AUC was 0.957 with a p-value of less than 0.001.

Sensitivity, specificity, positive predictive values (PPV), and likelihood ratios (LR) were calculated for each ETCO_2_ cut off point for all infants as shown in Table 2. An ETCO_2_ value between 17 and 18 mmHg had the highest sensitivity and specificity for return of a HR > 60 bpm for all infants. A positive likelihood ratio of 2.93 and 3.20 was found for ETCO_2_ values of 17 mmHg and 18 mmHg respectively. The PPV was 0.876 for an ETCO_2_ of 17 mmHg and 0.885 for an ETCO_2_ of 18 mmHg. The time gap between ETCO_2_ reaching 17 mmHg and then 18 mmHg with the corresponding heart rate was not measured. The area under the curve for the ROC curve for all infants is 0.835 with a p-value of less than 0.001 (see Fig. 1). Fig. 2 is the ROC curve that represents the sensitivity and specificity for return of HR > 60 bpm in those infants with the potential for decreased pulmonary blood flow.

**Table 2:** Sensitivity, specificity, positive predictive values and likelihood ratios for ETCO_2_ cutoffs for all infants as well as those with decreased pulmonary blood flow.

ETCO_2_ Cutoffs (mmHg)	Sensitivity	Specificity	1-Specificity	PPV	LR
All infants					
≥12	0.952	0.426	0.574	0.799	0.113
≥13	0.909	0.516	0.484	0.819	0.176
≥14	0.855	0.594	0.406	0.835	0.244
≥15	0.836	0.639	0.361	0.848	0.257
≥16	0.807	0.690	0.310	0.862	0.280
≥17	0.777	0.735	0.265	0.876	0.303
≥18	0.764	0.761	0.239	0.885	0.310
≥19	0.721	0.819	0.181	0.905	0.341
≥20	0.697	0.832	0.168	0.909	0.364
Infants with decreased pulmonary blood flow					
≥12	1.00	0.437	0.563	0.875	0.000
≥13	0.984	0.531	0.469	0.879	0.030
≥14	0.984	0.625	0.375	0.912	0.026
≥15	0.984	0.719	0.281	0.932	0.022
≥16	0.976	0.812	0.188	0.953	0.030
≥17	0.960	0.875	0.125	0.968	0.046
≥18	0.952	0.875	0.125	0.968	0.055
≥19	0.944	0.937	0.063	0.983	0.060
≥20	0.929	0.937	0.063	0.983	0.076

PPV = Positive predictive value; LR = Likelihood ratio.

## 4 Discussion

Our patient population included infants with a median age of 30 days that were in the PICU and the CVICU, with the majority of these infants having cardiac lesions. We corrected for low pulmonary blood flow for this reason and found that the ETCO_2_ number of 17 mmHg was not different for that group when analyzed separately. The results of this study support the use of ETCO_2_ monitoring during uninterrupted cardiac compressions to help predict when HR > 60 bpm has occurred and thus limit interruptions in compressions for auscultation. The difference in ROSC for the 18/21 patients in the potential for decreased pulmonary circulation group (85%) versus the 19/28 patients (68%) with normal pulmonary circulation was not different.

Cardiac compression methods achieve only a fraction (∼30%) of native perfusion even under the best of circumstances [21, 22]; however, with excellent CPR technique, preferential perfusion of the heart and brain during cardiopulmonary resuscitation (CPR) can result in greater than 50% of normal myocardial and cerebral blood flow [23, 24, 25]. When compressions are paused, the diastolic blood pressure (and thus the coronary perfusion pressure) generated from repetitive compressions is diminished and must be reestablished when compressions are reinitiated. Optimization of cardiac compressions and limiting interruptions might improve outcomes for infants who require CPR.

Three reports describe ETCO_2_ patterns during asphyxial arrest in animal models. Bhende *et al* [26] asphyxiated 3–6 month old dogs until cardiopulmonary arrest occurred. ETCO_2_ levels were initially elevated, decreased to low normal levels with ventilation, and increased to near-normal levels with ROSC. Berg *et al* [27] resuscitated ventricular fibrillated (VF) pediatric pigs or asphyxiated pediatric pigs after 15 min or 10 min of cardiac arrest respectively. Initial ETCO_2_ was noted to be high (91 ± 20 mmHg) in the asphyxiated pig model versus 34 ± 14 mmHg in the VF group. ETCO_2_ progressively decreased with each breath given but did not correlate with ROSC until one minute. Chalak *et al* [28] evaluated use of ETCO_2_ to predict ROSC in an asphyxiated asystolic neonatal piglet model where the animals had already undergone transition from fetal physiology. ETCO_2_ values were quite high during asphyxiation and then fell precipitously to zero or near zero mmHg during positive pressure ventilation following asystole. An ETCO_2_ cut-off value of 14 mmHg was the most sensitive ETCO_2_ value with the least false positives based on an ROC curve generated using HR > 60 as a positive result [28]. This study also shows that once the critical ETCO_2_ is reached (in this study 14) that heart rate rapidly increases.

A recent study by Berg et al. examined end tidal CO_2_ as a predictor of survival and found that a mean ETCO_2_ > 20 mmHg was not associated with improved survival or ROSC in the pediatric population. This study did not specifically look at how HR correlated with ETCO_2_ at a single point in time but at the overall mean [29]. Berg et al. results are in contrast to what has been found in adults where ETCO_2_ > 20 mmHg predicts ROSC while ETCO_2_ <10 mmHg after 20 minutes is associated with only 0.5% likelihood of ROSC [30]. Our study is the first to note ETCO_2_ patterns in relation to HR > 60 bpm in a cohort of infants undergoing CPR.

The American Academy of Pediatrics/AHA Neonatal Resuscitation Program (NRP) resuscitation guidelines recommend chest compressions for HR < 60 bpm despite 30 seconds of effective ventilation via an alternative airway (laryngeal mask or intubation) [1, 31]. Compressions are continued until an audible HR > 60 bpm is achieved. The latest NRP guidelines recommend using a cardiac monitor to determine heart rate during CPR; however, NRP continues to recommend pausing every 60 seconds to auscultate for heart rate to confirm when ROSC (HR > 60 bpm) has been achieved [1, 31]. Chest compressions are initiated in a 3:1 chest compression to ventilation ratio. However, a recent neonatal study suggests that it can take up to 17 seconds for providers to complete the heart rate assessment [32]. Cardiac monitoring can pick up pulseless electrical activity that can be mistaken for a functional heart rate, with auscultation being the gold standard [1]. ETCO_2_ values in this instance will be very low providing confirmation that the heart rate is inadequate.

As seen in our study, ETCO_2_ can also assess when and if HR > 60 bpm actually occurs. In the PALS algorithm used in this cohort of patients, chest compressions are started immediately upon finding asystole [19, 20]. However, with bradycardia (HR < 60 bpm), CPR is started if the infant has poor perfusion or lack of pulses after supplying oxygen and ventilation. Chest compressions are initiated in a 15:2 chest compression to ventilation ratio but once the child is intubated there is no stopping of chest compressions to deliver breaths [19, 20]; however, there are still interruptions to check for pulse every 2 minutes and for adequacy of breaths.

There are several limitations to our study. It is a retrospective chart review with the inherent bias of such a design. The resuscitations occurred in the PICU and CVICU which may not apply precisely to all infants, particularly newborns in the delivery room who are still undergoing transitional physiology. Again it should be noted that ETCO_2_ was correlated to heart rate with each specific value, the trend was not evaluated in each patient given the small sample size. Our study includes older infants that were resuscitated via PALs who already transitioned from fetal to neonatal circulation and included a high percentage of infants with cardiac lesions who are at higher risk for pulseless electrical activity even with heart rate >60 bpm. We also do not have ability to determine whether minute ventilation changed at all during CPR or when a pulse was obtained. Again this study may still be relevant for all neonates since the optimal ETCO_2_ cutoff was found to be similar even for infants with the potential for decreased pulmonary blood flow. It should be noted that the Area Under the Curve (AUC) was higher (0.957) in the group with potential for decreased pulmonary blood flow. There were more paired HR and ETCO_2_ that were both zero in these infants. This likely decreased the sensitivity and specificity for each ETCO_2_ value.

The current data may be more relevant to infants receiving CPR in the NICU or PICU than to those in the delivery room since these infants have already made the transition from in utero life. However, it may also be relevant to those infants in the delivery room that have elevated pulmonary pressures since this was not found to impact the threshold ETCO_2_ in this study. The confounding variable would be infants (especially preterm) that are unable to adequately make the transition quickly which would affect one's ability to adequately ventilate the lungs. A recent study of colorimetric ETCO_2_ detector in the delivery room of infants with HR < 100 bpm did find a correlation between gold color change (indicating CO_2_ detection) and increase in heart rate as we did in our study [33]. The median heart rate prior to gold color change was 75 bpm and increased to 136 bpm 36 seconds after gold color change. The colorimetric ETCO_2_ detector used in this particular study changes color when the ETCO_2_ is ≥ 15 mmHg which is very similar to the ETCO_2_ of 17 mmHg found in the current study even though NRP was used for their resuscitation.

Our study provides clinical information regarding ETCO_2_ values and HR > 60 bpm in infants during CPR. ETCO_2_ capnography provides a continuous, non-invasive alternative to eliminate frequent pauses auscultation of heart rate. In post-transitioned infants with cardiovascular collapse, achieving an ETCO_2_ value of at least 17 mmHg signals HR > 60 bpm. Such information is critical in order to design a future randomized clinical trial to determine if ETCO_2_ monitoring during resuscitation can reduce time to ROSC and subsequent morbidities compared to interrupting CPR and thus perfusion every 1–2 minutes to auscultate for return of heart rate.

## Declarations

### Author contribution statement

Christina N Stine: Conceived and designed the experiments; Analyzed and interpreted the data; Contributed reagents, materials, analysis tools or data; Wrote the paper.

Josh Koch, Myra H Wyckoff: Conceived and designed the experiments; Analyzed and interpreted the data; Contributed reagents, materials, analysis tools or data.

L. Steven Brown: Analyzed and interpreted the data.

Lina Chalak, Vishal Kapadia: Analyzed and interpreted the data; Contributed reagents, materials, analysis tools or data.

### Funding statement

This research did not receive any specific grant from funding agencies in the public, commercial, or not-for-profit sectors.

### Competing interest statement

The authors declare no conflict of interest.

### Additional information

No additional information is available for this paper.
